# A New Feature Set for Texture-Based Classification of Remotely Sensed Images in a Quantum Framework

**DOI:** 10.3390/jimaging12040149

**Published:** 2026-03-30

**Authors:** Archana G. Pai, Koushikey Chhapariya, Krishna M. Buddhiraju, Surya S. Durbha

**Affiliations:** 1 Centre of Studies in Resources Engineering, Indian Institute of Technology Bombay, Mumbai 400076, India; chhkoushikey@gmail.com (K.C.); bkmohan@csre.iitb.ac.in (K.M.B.); 2 MCA Department, Veermata Jijabai Technological Institute, Mumbai 400019, India

**Keywords:** remote sensing, texture classification, gray-level co-occurrence matrix, support vector machine, singular value decomposition, quantum machine learning

## Abstract

Texture feature extraction plays a crucial role in land-use and land-cover (LULC) classification for the remotely sensed images. However, when these images are quantized to a limited number of gray levels to reduce data volume or noise, conventional texture descriptors often lose discriminative power. This study investigates singular values of the gray-level co-occurrence matrix (GLCM) as novel texture features for image classification, with local binary pattern (LBP), complete LBP (CLBP) statistics, and original GLCM features proposed by Haralick et al. for comparison. Under coarse quantization, texture descriptors of LBP and its variants, which encode micro-texture, lose detail, whereas GLCM, which encodes macro-texture, retains structural co-occurrence patterns. This study thus proposes a new feature set, namely the Singular Values of the gray-level co-occurrence matrix (SVGM), for texture discrimination. Experimental analysis indicates SVGM achieves higher class separability by preserving dominant spatial structure while suppressing noise and redundancy. Quantitative evaluation using classical SVMs with multiple kernels, quantum learning models with different kernels, and neural baselines (ANN and 1D-CNN) further shows that SVGM consistently improves classification performance. Within our tested models, quantum kernel SVMs are competitive and achieve the best results on some datasets, while classical models perform best on others.

## 1. Introduction

Texture, with its visual representation, has been recognized as a fundamental element in image understanding, providing insights into the spatial arrangement and structural variations of natural and man-made surfaces. By utilizing spatial context to identify surface roughness and regularity, they are capable of modeling quantitative descriptors to capture local patterns, structural variation, and statistical dependencies that cannot be captured by spectral or intensity information alone. Such approaches have demonstrated strong effectiveness across diverse application domains, like image retrieval [1], biomedicine [2,3], fault detection [4,5], medical analysis [6,7], environmental resource management [8], and remote sensing [9]. As the complexity and dimensionality of modern datasets continue to grow, texture-based approaches offer a powerful pathway for capturing subtle spatial patterns that are otherwise difficult to model with traditional spectral or geometric features alone. In this work, we model spatial relationships using handcrafted descriptors to better distinguish visually similar yet structurally distinct classes, especially when intra-class variability is high, and class boundaries are nonlinear.

The emergence of deep learning has enabled powerful end-to-end classification pipelines that learn hierarchical texture representations directly from raw imagery. These models have demonstrated superior performance in capturing multi-scale, rotation-invariant, and nonlinear texture signatures, especially in large-scale, data-rich environments. However, the success of these classifiers often depends on large annotated datasets and extensive computational resources. Achieving these conditions in an operational remote sensing setting presents significant challenges [10]. This research is not focused on competing with the state of the art in texture recognition. Rather, it is about designing a simple architecture which enables faster feature extraction, reducing computational cost and increasing the texture feature stability using handcrafted feature descriptors in low-quantized images.

Success of texture analysis depends on careful consideration of factors such as image resolution [11,12,13], spatial heterogeneity, the choice of texture features [14,15], the effectiveness of feature selection methods [14], and the suitability of the classification algorithm [16] for the specific application and dataset. The progress of texture analysis in remote sensing is discussed in [17]. In the literature, texture classification approaches can be divided into four main categories. They are as follows: Statistical, structural, model, and transformation-based methods [18,19]. In [14,15], valuable insights into the effectiveness of different methods for capturing and analyzing textures, along with a review of various texture feature extraction techniques and their advantages and disadvantages, are discussed. The study in [3] provides a detailed analysis of the visual perception of textures. Structural approaches are not appropriate for textures with a high degree of randomness, as in remotely sensed images [14]. Statistical approaches of texture are often found to be better descriptors than geometrical and model-based methods in processing and interpretation of remote sensing images, which are a mixture of different textures without clear texture boundaries and a high degree of randomness [20].

In the literature, statistical approaches such as the GLCM, LBP, and CLBP are widely used to interpret image textures. GLCM-based texture analysis, introduced in [21], is a simple and thoroughly studied statistical texture analysis method [22]. Its effectiveness in capturing macro textures [23] makes it a benchmark approach in texture feature extraction with an extensive list of applications in medical imaging [2,24], crop monitoring [9], and remote sensing [25,26]. In [27], GLCM features were used for the classification of images from the Brodatz dataset. Dimensionality of the co-occurrence matrix depends on the number of gray levels used to define the image. Computational cost and time increase quadratically with the increase in the number of gray levels used [28] and the window size used for feature extraction [11]. Using directional information improves classification accuracy, increasing memory requirements, especially when computed across several directions and distances [29]. In this experimental work, we aimed to address issues related to selecting GLCM features and the high dimensionality of co-occurrence matrices.

Singular Value Decomposition (SVD) is a powerful technique commonly used for dimensionality reduction, with applications in image processing, particularly for image compression. There are several instances where this technique is applied for classification [30], fault detection [31,32], image retrieval [1], surface roughness measurement [33], color to grey scale conversion [34], and texture analysis. SVD, when used for texture analysis, offers efficient and robust texture features [35] and is least affected by noise [36]. In literature, a few researchers have used singular values [33,35,37] of images as texture features, while others have used eigenvectors as features [38]. Applying SVD to an image reveals fundamental information, such as edges and textures [39]. The singular values thus generated capture the underlying structure and energy distribution of the image. In this research work, we used SVD to compress the co-occurrence matrix of GLCM into dominant spatial co-occurrence patterns, which are stable, low-rank components that reduce the impact of noise and illumination variations while preserving the most meaningful texture information. Singular values capture the independent texture modes and their strengths.

Recent advances in quantum machine learning (QML) have introduced a new computational paradigm for image classification. Unlike classical kernels, which operate in fixed-dimensional feature spaces, quantum kernels leverage high-dimensional Hilbert spaces [40] generated through parameterized quantum circuits to capture complex nonlinear relationships that may be difficult to represent using conventional methods [41,42,43]. In [44], Quantum SVM (QSVM) was coined for the first time and demonstrated that it reduces the complexity to O(log(MN)), compared to the O(poly(MN)) complexity of classical methods, where N represents the dimensionality of the data, and M represents the number of training samples. The links between kernel methods and quantum computing are explored in [45], building the foundation for kernel-based QML. QSVMs are currently applied across a broad range of domains, including finance [46], healthcare [47,48], remote sensing [41,49], and environmental monitoring [8]. Using IBM’s circuit-based [50] paradigm, in [51], QSVM was proposed as a multiclass classifier; in [52,53] as a texture-based binary and multiclass classifier, respectively. Using the quantum annealing paradigm [54], QSVM was proposed in [43,55] for binary and multiclass classification of remotely sensed images. redThe status of quantum computers (QCs) for earth observation is presented in [56], analyzing their potential limitations and applications in quantum learning models for satellite data, while considering the persistent challenges of achieving quantum advantage. A QSVM-based image classification approach is proposed in [47], using features extracted from ResNet-10 combined with angle encoding, and its effectiveness is evaluated across four datasets. Although QC is still in the early stages of development, in terms of the number of qubits, quantum volume, and error mitigation, it has significant potential to advance the capabilities of remote sensing image analysis [57]. In this study, we used QSVM for texture-based classification of the newly proposed SVGM features.

All the above-mentioned texture feature extraction methods have shown fulfilling results in one way or another, but some questions still need definite answers.

(a) Dimensionality of the co-occurrence matrix depends on the number of intensity levels used to represent the image. How can the information found in the high-dimensional co-occurrence matrix be represented in a reduced space? Such that it enables efficient image comparison with reduced computational time and resource requirements for low-quantized images?
(b) The co-occurrence matrix is highly dependent on intensity changes. How can the effects of intensity changes within these high-dimensional matrices be stabilized while preserving the textural information content found in high dimensional co-occurrence matrix?
(c) To what extent can quantum kernel methods enhance texture discrimination in a low-quantized image, particularly in scenarios with nonlinear class boundaries or limited training samples?

This article proposes a new texture feature set, called Singular values of GLCM (SVGM), which answers the first two questions mentioned above. Using SVD Decomposition on the high-dimensional co-occurrence matrix of the GLCM helps isolate the most informative spatial co-occurrence structures. The resulting singular values represent a compact, semantically meaningful feature set. First, reduce the number of gray levels in the image using multi-level OTSU thresholding. Then, for each patch of the thresholded image, calculate the directional GLCM. The singular values of the SVD decomposition of the GLCM form the proposed SVGM features.

To answer the third question, the efficiency of the proposed texture feature, SVGM, was tested using QSVM with three different quantum kernels. Quantum computing operates on multiple data points simultaneously through the principle of superposition, which can potentially reduce time complexity. Quantum kernels leverage the high-dimensional feature space induced by quantum states to compute inner products, providing an exponential speedup and improving classification accuracy.

The main contribution of this research work can thus be summarized as follows:

- A new feature set, SVGM, is proposed to represent the spatial information present in GLCM as a compact set enabling dominant texture patterns that remain stable for small intensity changes in a low-quantized image.
- The effectiveness of the proposed feature set is demonstrated experimentally on four different datasets, and using five different kernels - two classical and three quantum. We experimentally found that a compact set of values can differentiate between different texture patches, especially under various conditions of low quantization, high texture variability, or limited training data.
- This is the first time a texture analysis is carried out in a quantum framework.

The rest of this article is organized as follows. Section 2 explains the proposed model and the various techniques used for feature extraction in this research work. Section 3 presents the experimental results using the proposed feature set, along with their discussion in Section 4. Finally, Section 5 concludes this article with a future scope to extend this work.

## 2. Materials and Methods

In this section, we outline the proposed methodology for creating a new set of texture features, referred to as SVGM. In Section 2.1, we provide a brief description of the dataset considered. Furthermore, Section 2.2 explains the workflow and algorithm of the proposed methodology.

### 2.1. Dataset

**Figure 1:**
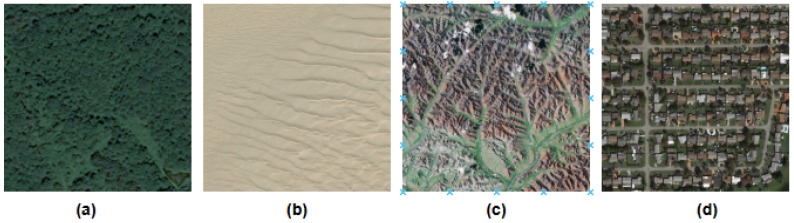
Samples of different classes (**a**) forest, (**b**) desert, (**c**) mountain, (**d**) residential from the AID datasets.

Aerial Image Dataset (AID) [58] comprises 10,000 high-resolution images, each sized at 600 × 600 pixels, spanning 30 different classes with 200 to 400 images per class. These images are sourced from various locations around the world, captured at different times, seasons, and imaging conditions using different sensors as shown in Figure 1. The dataset also features multi-resolution images, increasing both intra-class and inter-class diversity. This diversity presents significant challenges for scene classification, as it includes scenes of varying sizes and orientations, captured at different flying altitudes and directions, and under different solar elevation angles.

**Figure 2:**
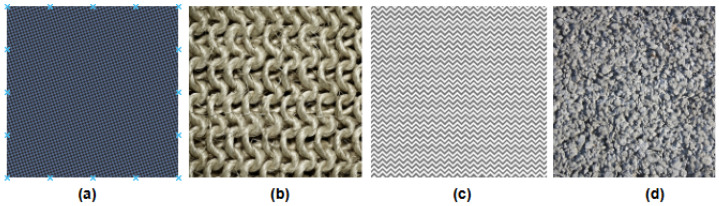
Samples of different classes from the DTD datasets: (**a**) chequered, (**b**) woven, (**c**) zigzagged, (**d**) porous.

Describable Textures Dataset (DTD) [59] is a widely used dataset for evaluating texture recognition and segmentation. It comprises 5640 texture images, divided into 47 describable texture categories, which are inspired by human perception of textures. The images in the dataset are collected from diverse sources and exhibit high variability in terms of scale, viewpoint, and illumination, making the dataset challenging and realistic for texture analysis tasks, as shown in Figure 2. Its unique focus on describable attributes enables researchers to develop and evaluate models that align with human perception, bridging the gap between computational and cognitive understanding of textures.

**Figure 3:**
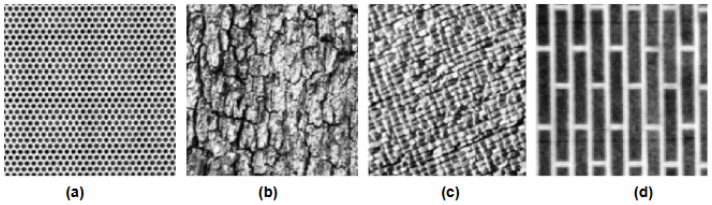
Samples of different classes from the Brodatz datasets: (**a**) holearray, (**b**) bark, (**c**) raffia, (**d**) brickwall.

Brodatz dataset [60,61] database stands as a foundational resource in the realm of image processing and computer vision for texture analysis. The USC-SIPI team compiled this dataset, which contains four volumes based on the basic characteristics of the pictures. Images in each volume are of various sizes, such as 256 × 256 pixels, 512 × 512 pixels, or 1024 × 1024 pixels. All images are either 8-bit/pixel black and white images or 24-bit/pixel color images. The sample of different classes is shown in Figure 3.

**Figure 4:**
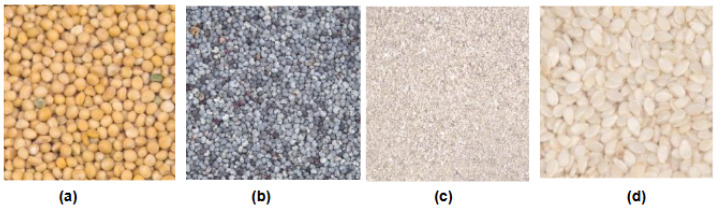
Samples of different classes from the ALOT datasets: (**a**) grain, (**b**) pebbles, (**c**) sand, (**d**) til.

Amsterdam Library of Textures (ALOT) dataset [62] is a collection of 250 color images of rough textures. To capture sensory variations in the recordings, images were systematically captured at different viewing angles, illumination angles, and illumination colors for each material. The collection comprises linear mixtures of 12 materials, yielding over 27,500 images, with 100 images recorded for each material. A few sample classes are shown in Figure 4. ALOT extends the CURET dataset by including a greater number of materials, while also enhancing image resolution and color quality.

### 2.2. Proposed Framework

**Figure 5:**
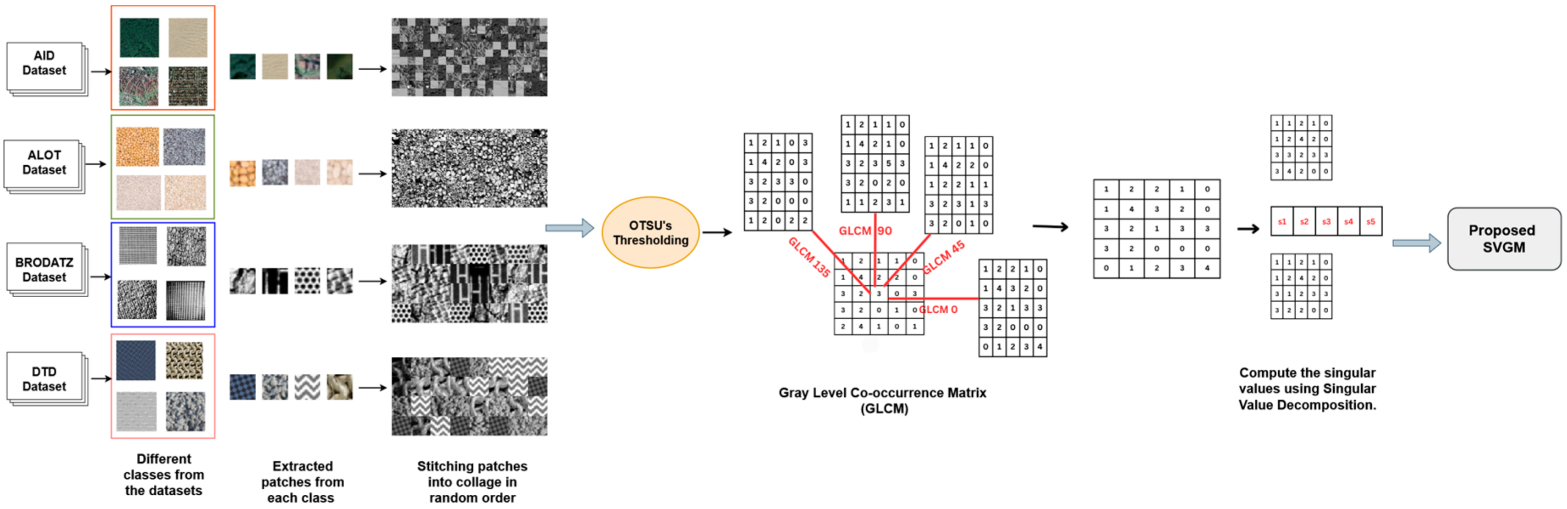
Workflow of the proposed SVGM texture descriptor from input texture patches to GLCM-based SVGM feature vectors for multiclass texture classification.

In this study, we introduce SVGM, a novel set of texture features aimed at improving patch-based multiclass texture classification in remote sensing images. In patch-based classification, an image is divided into smaller, fixed-size regions or patches, and each patch is individually analyzed and classified based on its textural characteristics. An overview of the proposed SVGM workflow is presented in Figure 5.

In the proposed framework, SVGM features are extracted in a patch-based manner from multiple texture datasets (AID, ALOT, Brodatz, and DTD). After collecting the images, each RGB image is first converted to grayscale. The grayscale images are divided into fixed-size non-overlapping patches to build a diverse pool of texture samples. For computational convenience, we assemble randomly selected patches into a collage only to estimate Otsu-based quantization levels. This collage preserves local texture characteristics while reducing the influence of absolute spatial location and providing a richer mixture of texture patterns within a single image. The quantized image is then used to collect SVGM features from each patch. The classifier operates on patch-level feature vectors. This step is used to enforce a consistent gray-level discretization before GLCM computation and does not combine patches during feature extraction or classification.

The collage image is then segmented using four-level Otsu thresholding [63], producing a low quantized representation that enhances dominant intensity structures. It also enforces a common intensity scale across all patches. From this thresholded collage, gray-level co-occurrence matrices (GLCMs) are computed for each patch with d = 1, typically in four principal directions (0°, 45°, 90°, and 135°) to capture direction-dependent second-order spatial statistics. GLCM texture descriptors included angular second moment (ASM), contrast, dissimilarity, correlation, and standard deviation. This subset summarizes the most important texture information in a clear and non-overlapping way. Together, they express the texture in terms of uniformity (smooth or textured), local variation, the relationship between neighbouring pixels, and the overall spread of intensity values. The 4-directional GLCMs are then aggregated, and GLCM texture descriptors are computed to give a 5-dimensional GLCM feature vector.

SVD is applied to the averaged GLCM, and a reduced set of five singular values is retained. This smaller set of values makes it easier to analyze and compare texture features across different regions of an image. Singular values are invariant to rotation and scaling transformations, making them robust features for texture analysis. This invariance is crucial in remote sensing for land cover classification, where images can be captured at different angles and scales. These singular values form our proposed set of SVGM features, which are then used as inputs to the multiclass texture classification stage. A brief algorithm for generating the proposed SVGM features is presented in Algorithm 1, followed by the mathematical representation for feature extraction.
**Algorithm 1** SVGM Texture Feature Extraction  1:**Input:** Set of labeled images $\left\{I_{1},I_{2},\ldots ,I_{N}\right\}$, patch size *p*, threshold levels *t*  2:**Output:** Feature matrix *F* for classification  3:**for** each image $I_{i}$ in dataset **do**  4:      Divide $I_{i}$ into non-overlapping patches of size p×p  5:      Store all patches in set P  6:**end for**  7:Shuffle P randomly  8:Construct collage image *C* by arranging patches from P in grid layout  9:Apply Otsu thresholding to *C* to obtain $C_{t}$ with *t* levels10:Initialize feature matrix F←∅11:**for** each patch $P_{j}$ in $C_{t}$ **do**12:      Compute GLCM matrices $G_{0}$, $G_{45}$, $G_{90}$, $G_{135}$ for directions 0°, 45°, 90°, 135° and d=113:      Aggregate GLCMs: $G\leftarrow \frac{1}{4}\left(G_{0}+G_{45}+G_{90}+G_{135}\right)$14:      Perform SVD: $SVD\left(G\right)=U\Sigma V^{T}$15:      Extract top-*t* singular values from Σ as feature vector $f_{j}$16:      Append $f_{j}$ to feature matrix *F*17:**end for**18:**Return** *F*

### 2.3. Mathematical Computation of SVGM Features

Let I be the input thresholded collage image, $P_{i}\in R^{p\times p}$ be the *i*th patch extracted from I. $G_{i}^{\theta }$ be the GLCM of $P_{i}$ computed in direction θ∈{0°,45°,90°,135°}

1. GLCM calculation: For each patch $P_{i},$ of the input thresholded collage image, if $G_{i}^{\theta {}^{\circ}}$ denotes the GLCM of patch $P_{i}$ in a direction θ, a particular element $G_{i}^{\theta {}^{\circ}}\left(i,j\right)$ is defined as

$$G_{i}^{\theta {}^{\circ}}\left(i,j\right)=\#P_{i}\left(m,n\right)=i,P_{i}\left(m+dr,n+ds\right)=j$$

where dr and ds being the displacements from a pixel at location (m,n) to reach the neighboring position (m+dr,n+ds) in the direction of θ.

2. GLCM Aggregation: For each patch $P_{i},$ compute the average of $G_{i}^{0{}^{\circ}}$, $G_{i}^{45{}^{\circ}}$, $G_{i}^{90{}^{\circ}}$, and $\;G_{i}^{135{}^{\circ}}$ directional GLCM corresponding to the patch $P_{i}$:

$$G_{avg}=\frac{1}{4}\left(G_{i}^{0{}^{\circ}}+G_{i}^{45{}^{\circ}}+G_{i}^{90{}^{\circ}}+G_{i}^{135{}^{\circ}}\right)$$

3. SVD Decomposition: Apply Singular Value Decomposition to the aggregated GLCM, $G_{avg}$:

$$SVD\left(G_{avg}\right)=U_{i}\Sigma_{i}V_{i}^{T}$$

4. Feature Extraction: Extract the singular values resulted from $\Sigma_{i}$ of $SVD(G_{avg})$ for the patch $P_{i}$. And these singular values form the feature vector, SVGM:

$$SVGM=\Sigma_{i}=\sigma_{i1},\sigma_{i2},\ldots ,\sigma_{ik}$$

The distribution of SVGM follows a pattern and varies from texture to texture, facilitating effective discrimination within texture patches. Larger values of SVGM correspond to the dominant patterns in the data, indicating that they represent the most prominent and repetitive spatial relationships within the texture of the image. These dominant patterns can include regular repetitions, uniformity, or specific directional textures, which are indicative of certain land cover types. The forest class had periodic texture, and it can be seen that it is represented using the first two singular values. For the desert class, it had a smooth texture, which was represented by a single dominant singular value. However, the residential and mountain classes have complex textures and are therefore represented using several comparable singular values.

## 3. Results

The following section discusses the experimental setup and the factors that influence the model’s performance, including different parameters and model configurations. Furthermore, the performance of the proposed feature set is evaluated by calculating various qualitative and quantitative metrics.

### 3.1. Experimental Configuration and Parameter Settings

In this research, experiments are conducted on a computer system equipped with an Intel(R) Core(TM) i7-7700 CPU @ 3.60 GHz and 16.0 GB of installed RAM. The software environment consists of a 64-bit operating system with Windows 10 Pro for Workstations. To execute these experiments, we used the Anaconda framework with Python 3.8. We utilized various libraries, including NumPy (ver 1.6), Seaborn (ver 0.13.2), Matplotlib (ver 3.10), scikit-image (ver 0.26), scikit-learn (ver 1.8), and OpenCV (ver 4.13). For this image classification experiment, we utilized IBM’s Qiskit [50] package. Quantum kernels were generated using Qiskit, and the ZZ [64] feature map was used to encode the data into a quantum state.

In quantum computing, entanglement refers to the connection between qubits [50]. QLinear, QCircular, and QFull represent quantum kernels with linear, circular, and full entanglement, respectively. In linear entanglement, the 1st qubit is connected to the 2nd qubit; the 2nd qubit is connected to the 3rd qubit, and so on. Circular entanglement is a special case of linear entanglement with an additional connection where the last qubit is connected to the 1st qubit. In full entanglement, every qubit is connected to every other qubit.

The various other parameters used in this research include image patches of size 25 and approximately 200 image patches for each class, resulting in around 800 patches per dataset. Later, we split the feature vector collection into 70% training and 30% testing. For this experiment, we used three trees for the Random Forest classifier. Our ANN consisted of two fully connected layers with six units each and ReLU activations, followed by a four-unit softmax output layer, and was trained for 70 epochs using the Adam optimizer with categorical cross-entropy as the loss function. The 1D-CNN consisted of two convolutional layers with 16 and 32 filters, respectively, followed by a 32-neuron dense layer with Leaky ReLU as activation and a four-unit softmax output layer, and was trained for 50 epochs using the Adam optimizer with categorical cross-entropy as loss function.

### 3.2. Comparison with State-of-the-Art Methods

In this research work, we compared the performance of our proposed SVGM features to that of the Haralick GLCM [21], LBP features [65,66] and CLBP [67]. We chose these methods to compare with the proposed SVGM because they are widely used, well-established texture descriptors that represent complementary second-order co-occurrence and local pattern baselines. The sign component of CLBP (CLBP-S) is very close to standard LBP, since both are built from the same idea of comparing each neighbour to the center pixel and forming a binary pattern [68]. So no new additional information was added, especially when images are coarsely quantized, as many neighbouring pixels end up with the same intensity. In contrast, CLBP-M focuses on how great the local changes $|I_{n}-I_{c}|$ are with $I_{n}$ representing the neighboring pixel and $I_{c}$ representing the center pixel. This helps separate smooth areas from more textured or edge-rich regions, even with limited gray levels.

**Figure 6:**
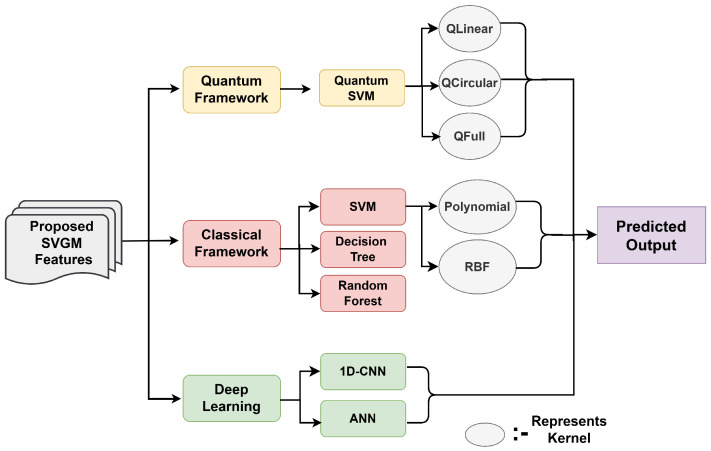
Classification architecture illustrating various classifiers used to evaluate the performance of the proposed SVGM feature set.

We compare performance across three categories: the quantum framework, the classical framework, and the deep learning framework, as shown in Figure 6. In the quantum framework, we evaluate QSVM models using three kernels: QLinear, QCircular, and QFull based on different entanglements as stated earlier. The classical framework includes traditional SVM with Polynomial and Radial Basis Function (RBF) kernels, along with Decision Tree and Random Forest classifiers. For the deep learning framework, we consider two architectures: a 1D-CNN and a fully connected ANN.

#### 3.2.1. Quantitative Evaluation

We have evaluated the performance of the proposed method using three standard evaluation metrics: overall accuracy (OA), average accuracy (AA) and kappa coefficient (K). The classification accuracies for all four datasets are computed and summarized in Table 1 for AID dataset, Table 2 for ALOT dataset, Table 3 for Brodatz dataset, and Table 4 for DTD dataset. For almost all of the cases, the newly proposed SVGM features outperformed the Harlick GLCM and LBP features irrespective of the computing (classical or quantum) framework. With the proposed SVGM features:

(a) For the AID dataset:
  (i) When compared to GLCM, the performance was enhanced between 1–4% across all kernels and classifiers except for RF, where it dropped by 0.5%.
  (ii) When compared to LBP, the performance was enhanced between 4–30% across all kernels and classifiers.
  (iii) When compared to CLBP, the performance was enhanced between 4–35% across all kernels and classifiers.
(b) For the ALOT dataset,
  (i) When compared to GLCM, the performance varied between 0.5–3% across all kernels and classifiers, except for RF, where it dropped by 0.5%.
  (ii) When compared to LBP, the performance was enhanced between 4–30% across all kernels and classifiers.
  (iii) When compared to CLBP, the performance was enhanced between 6–35% across all kernels and classifiers.
(c) For the Brodatz dataset:
  (i) When compared to GLCM, the performance was enhanced between 1–3% across all kernels and classifiers, except for QLinear and QCircular, where it reduced by 0.5%.
  (ii) When compared to LBP, the performance was enhanced between 1–13% across all kernels and classifiers.
  (iii) When compared to CLBP, the performance was enhanced between 2–15% across all kernels and classifiers.
(d) For the DTD dataset:
  (i) When compared to GLCM, the performance varied between 0–1% across all kernels and classifiers, except for QLinear, QCircular, and RF, where it dropped by 0.5%, and for 1D-CNN it dropped by 5%.
  (ii) When compared to LBP, the performance was enhanced between 3–21% across all kernels and classifiers.
  (iii) When compared to CLBP, the performance was enhanced between 2–18% across all kernels and classifiers.

On challenging texture benchmarks such as DTD, transfer learning with pre-trained CNNs (e.g., VGG/ResNet) has been shown to achieve strong performance. Our results should therefore be interpreted in the context of lightweight, feature-based baselines, while deep texture representations, e.g., [69], may yield higher accuracy.

#### 3.2.2. Qualitative Evaluation

**Figure 7:**
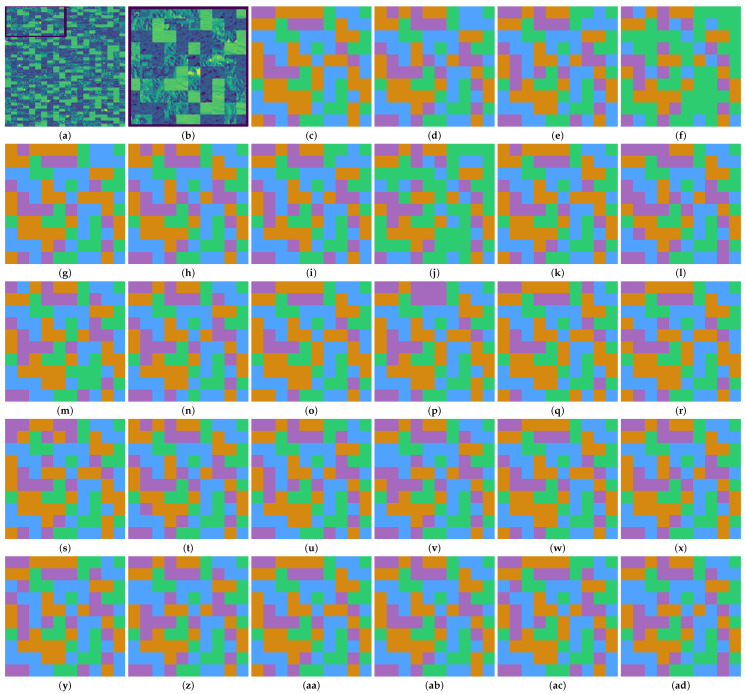

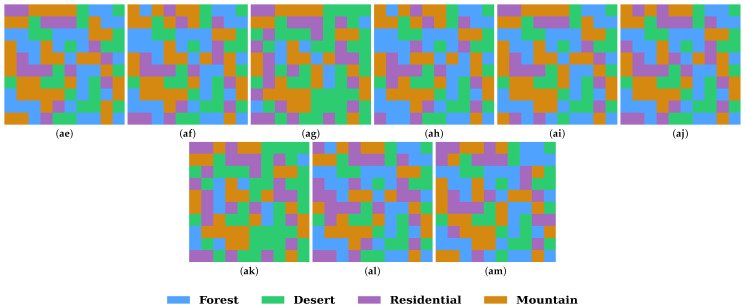
Classification maps produced by different classifiers trained using different feature sets like SVGM, GLCM, LBP and CLBP on the AID dataset. (**a**) Collage Image, (**b**) Zoomed sub-image (**c**) Ground Truth (**d**) GLCM Poly (**e**) LBP Poly (**f**) CLBP Poly (**g**) SVGM Poly (**h**) GLCM RBF (**i**) LBP RBF (**j**) CLBP RBF (**k**) SVGM RBF (**l**) GLCM DT (**m**) LBP DT (**n**) CLBP DT (**o**) SVGM DT (**p**) GLCM RF (**q**) LBP RF (**r**) CLBP RF (**s**) SVGM RF (**t**) GLCM QLinear (**u**) LBP QLinear (**v**) CLBP QLinear (**w**) SVGM QLinear (**x**) GLCM QCircular (**y**) LBP QCircular (**z**) CLBP QCircular (**aa**) SVGM QCircular (**ab**) GLCM QFull (**ac**) LBP QFull (**ad**) CLBP QFull (**ae**) SVGM QFull (**af**) GLCM 1D-ANN (**ag**) LBP 1D-ANN (**ah**) CLBP 1D-ANN (**ai**) SVGM 1D-ANN (**aj**) GLCM CNN (**ak**) LBP CNN (**al**) CLBP CNN (**am**) SVGM CNN.

The visual comparison of the proposed method with other methods, namely GLCM, LBP, and CLBP for the AID dataset, is shown in Figure 7. We include a zoomed-in view of the collage to improve the clarity and visualization of the results. The classification map indicates that all features performed well at identifying forest and desert classes. Our proposed texture feature, SVGM, has shown superior performance in classifying the residential and mountain classes when compared with GLCM, LBP, and CLBP features. Furthermore, the quantum linear kernel outperformed all other classifiers considered in this work. To maintain clarity and prevent information overload, we only present visualization results for the AID dataset, while the remaining classification results are included in Appendix A for reference.

**Figure 8:**
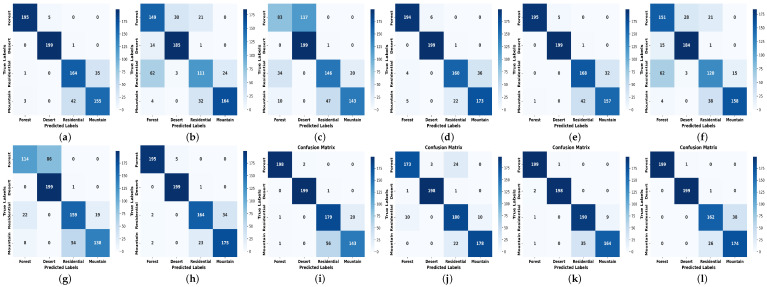

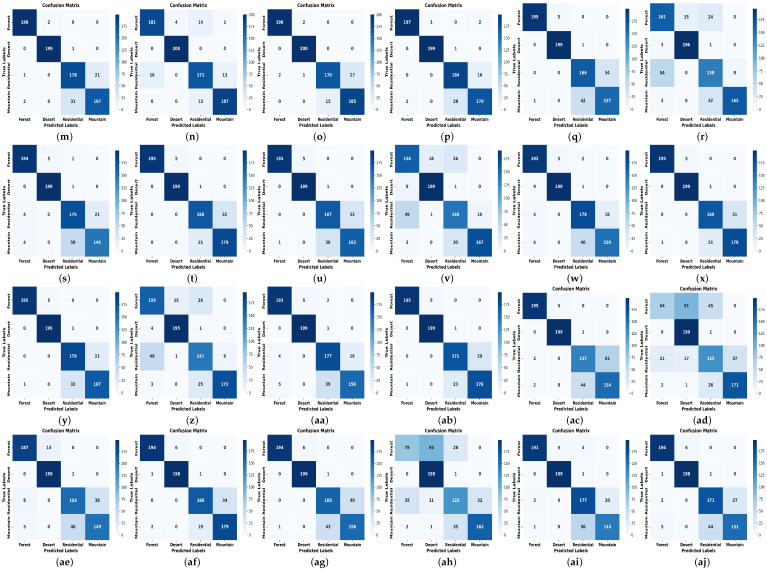
Confusion matrices depicting the performance of various classifiers on the AID dataset, illustrating the class-wise prediction performance of each model using four feature sets: SVGM, GLCM, LBP, and CLBP. (**a**) GLCM Poly (**b**) LBP Poly (**c**) CLBP Poly (**d**) SVGM Poly (**e**) GLCM RBF (**f**) LBP RBF (**g**) CLBP RBF (**h**) SVGM RBF (**i**) GLCM DT (**j**) LBP DT (**k**) CLBP DT (**l**) SVGM DT (**m**) GLCM RF (**n**) LBP RF (**o**) CLBP RF (**p**) SVGM RF (**q**) GLCM QLinear (**r**) LBP QLinear (**s**) CLBP QLinear (**t**) SVGM QLinear (**u**) GLCM QCircular (**v**) LBP QCircular (**w**) CLBP QCircular (**x**) SVGM QCircular (**y**) GLCM QFull (**z**) LBP QFull (**aa**) CLBP QFull (**ab**) SVGM QFull (**ac**) GLCM 1D-ANN (**ad**) LBP 1D-ANN (**ae**) CLBP 1D-ANN (**af**) SVGM 1D-ANN (**ag**) GLCM CNN (**ah**) LBP CNN (**ai**) CLBP CNN (**aj**) SVGM CNN.

We have also computed the confusion matrices for the AID dataset as shown in Figure 8. We used a confusion matrix as it provides a detailed breakdown of a classifier’s performance across all classes. Unlike overall accuracy, it shows how many samples were correctly classified and where misclassifications occurred. This helps identify which classes are being confused with one another, detect class imbalance issues, and evaluate the strengths and weaknesses of our proposed model at a more granular level.

### 3.3. Ablation Study

#### 3.3.1. Performance Evaluation Using Different Texture Patterns

**Figure 9:**
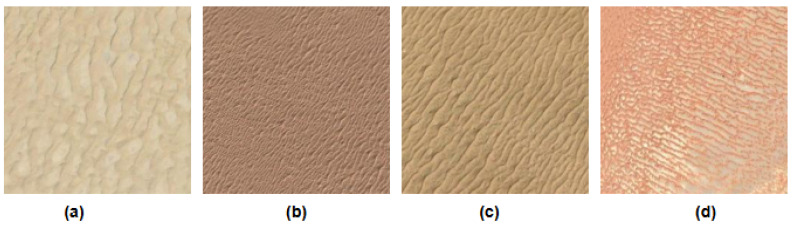
Samples of different classes from the Desert class of AID datasets: (**a**) desert1, (**b**) desert2, (**c**) desert3, (**d**) desert4.

We considered various patterns of desert texture as shown in the Figure 9 from the desert class of the AID dataset. We examined whether our proposed texture feature is capable of classifying these different textures. From the experiment, it was found that it is capable of classifying different textures, and the results of the classification are given in Table 5.

With our proposed SVGM features:

(i) When compared to GLCM, the performance varied between 3–5% across all kernels and classifiers.
(ii) When compared to LBP, the performance varied between 6–15% across all kernels and classifiers.
(iii) When compared to CLBP, the performance varied between 17–19% across all kernels and classifiers.

#### 3.3.2. Effect of Number of Image Patches

Here, we studied the effect of the number of image patches on the overall accuracy of classification. We varied the number of image patches per class from 100 to 250 with a step size of 50. Using our proposed SVGM features and other features, the accuracy improved as the number of image patches increased. We fixed the number of images per class to 200, as it provided us with optimal accuracy, as tabulated in the Table 6.

#### 3.3.3. Effect of the Window Size

The window size determines the area considered to accurately classify the central pixel. We have compared the effect of different window sizes on overall accuracy by varying them from 15 to 30, with a step size of 5, on the AID dataset. Based on the analysis tabulated in Table 7, we considered a window size of 25 for all datasets.

From the Table 7, it can be seen that SVGM performance improved with increasing window size.

#### 3.3.4. Effect of Number of Gray Levels

**Figure 10:**
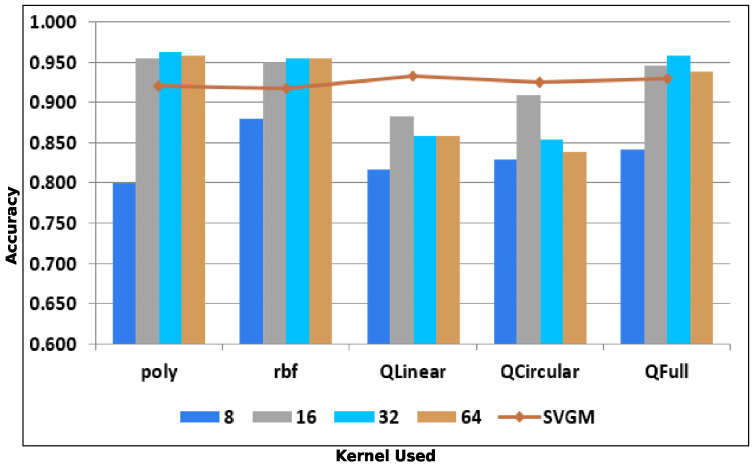
Effect of Number of Gray Levels.

In this experiment, we varied the number of gray levels used to represent the image and assessed their effect on the overall accuracy of various classification features. We represented the images using 8, 16, 32, and 64 gray levels and collected GLCM features. Despite the increase in gray levels, the overall accuracy of classification remained almost the same at around 95% for gray levels 16, 32, and 64 with poly, rbf, and QFull kernels. In contrast, our proposed SVGM features with only 5 gray levels gave us an overall accuracy of 91% for classical kernels and 94% for quantum kernels. This analysis can be visualized in Figure 10, where the line graph represents the accuracy obtained by our proposed SVGM features. LBP and CLBP work on binary thresholds and are very sensitive to the number of gray levels, except for a minor loss of local texture precision.

#### 3.3.5. Comparison of Computation Time

In this experiment, we varied the number of gray levels used to represent the images and the computational time required for calculating the features. We collected GLCM features from images represented using gray levels 8, 16, 32, and 64. The computational time required for extracting GLCM features was 588 ms, 797 ms, 990 ms, and 1772 ms, respectively. This demonstrates a significant increase in computational time for GLCM features with the increase in the number of gray levels. The computation time required for our proposed SVGM remained constant at 87 ms, for LBP was about 1500±60 ms, and for CLBP was about 1600±40 ms. This analysis is quantified in Table 8. Since we use the same classifiers and the same number of training/testing patches across all descriptors, each with small feature vectors, the time taken by the classification stage should be roughly the same for SVGM, Haralick-GLCM, LBP, and CLBP. As a result, the main computational difference comes from the feature-extraction step.

## 4. Discussion

The results of this research demonstrate the effectiveness of the texture-based representation for the classification of images involving complex spatial patterns based on low-quantized images. In this study, we compare the performance of our proposed SVGM feature set with LBP, CLBP (magnitude), and GLCM. CLBP mainly extends LBP by adding CLBP-S (Sign), CLBP-M (magnitude), and CLBP-C (center) to capture more information. In this research work, we have considered only CLBP-M, as it is more responsive to texture variations than CLBP-S and CLBP-C. CLBPs, magnitude component (CLBP-M) captures the difference in magnitude between the neighbors and the center pixel |In−Ic| so it focuses on how great these local changes are (flat vs. edge/rough) rather than only “up/down”. Even when intensities are coarse, the magnitude still separates zero change from non-zero change, which helps in differentiating textures. LBP is a descriptor of a micro pattern that is invariant to gray scale variations, can be represented using a smaller feature set, has low memory requirements, and is faster in computation. GLCM is a macro-pattern texture descriptor represented as a co-occurrence matrix whose size depends on the number of gray levels, and the Haralick features derived from it are often correlated. For an image with N gray levels, the GLCM has size N×N, so the memory requirement grows rapidly, proportional to $N^{2}$. In practice, this quadratic cost is further amplified because GLCMs are typically computed for multiple directions and inter-pixel distances, and for every patch used in the experiment. Since the matrix stores co-occurrence counts as integers, the memory required per GLCM is approximately $K\times N^{2}$ bytes when using `K’ byte integer storage.

Among the evaluated feature sets, our proposed SVGM features consistently yielded superior classification. This improvement can be attributed to SVD’s ability to extract the dominant texture [39] from GLCM matrices while suppressing noise and redundant information. The resulting singular values form a compact feature space, which remains stable under intensity variations and severe gray-level reduction. This work is validated through an experimental study over four different datasets. In this work, we modeled spatial relationships using handcrafted descriptors to distinguish visually similar yet structurally distinct classes. This work is aimed at designing a simple classifier that enables faster feature extraction with reduced computation and increases the texture feature stability in low-quantised images.

When the image was quantized, multiple pixel intensities were mapped to the same quantized bin, reducing differences between neighboring pixels. This directly resulted in a reduction in LBP code diversity, affecting the mean and variance of LBP codes and reducing the discriminative power of LBP features. Also, different histograms shared similar moments, reducing the discriminative power further and increasing confusion. The co-occurrence matrix of GLCM in low-quantized images is smaller, preserving pairwise spatial relationships between brightness bins and providing reliable statistics that are robust to moving windows. However, with granular textures, GLCM fails to capture the subtle texture variations and lacks the discriminative power. The SVD decomposition of these densely populated, smaller GLCM co-occurrence matrices is more stable, faster, and better at capturing consistent spatial patterns. The singular values of the resulting SVD decomposition capture dominant texture modes, resulting in compact, informative texture features.

Quantum kernel-based classifiers have shown a performance advantage over classical kernels, suggesting their ability to capture complex feature relationships in high-dimensional texture spaces. Class-wise analysis further reveals that homogeneous land cover types such as Forest and Desert were classified with high consistency across most classifiers, owing to their relatively uniform texture and spatial structure. In contrast, Residential and Mountain classes posed greater challenges due to their inherent heterogeneity, bright, sharp edges, and mixed spatial signatures. The improved delineation of these challenging classes using SVGM features indicates the importance of capturing higher-order spatial organization rather than relying solely on local or first-order descriptors.

Despite these promising results, the study has certain limitations. The capability of the proposed SVGM features in differentiating between objects with different scales, patterns, and orientations is limited to the patch size, fixed gray-level quantization, and a predefined set of texture features. Additionally, the current experiments were limited to ZZ feature maps, and further exploration of quantum feature maps may yield additional insights. It will be interesting to observe the efficiency of the model on a noisy dataset. We acknowledge that more recent texture representations, such as modern handcrafted variants and transfer-learning CNN features (e.g., VGG/ResNet-based deep texture models), can provide stronger baselines on challenging datasets. Extending the comparative evaluation to these recent methods using a unified, carefully tuned protocol will also be considered in future work.

## 5. Conclusions

This study evaluates the role of texture in image classification across a range of classical, deep learning, and quantum classification models in a low quantized images. We propose a new set of texture features, SVGM, to provide a compact, stable, and interpretable feature representation that enables efficient image comparison. SVGM are singular values produced by the SVD decomposition of GLCM co-occurrence matrices. By systematically evaluating the local and statistical features across LBP and CLBP, conventional GLCM statistics, the study demonstrates the role of mathematical transformations in enhancing texture discriminability across both homogeneous and heterogeneous land cover classes while reducing feature redundancy and computational complexity. This study highlights the adaptability of handcrafted texture features to diverse learning paradigms. It can serve as a strong foundation for both conventional classifiers and the emerging quantum learning frameworks. Overall, the proposed approach offers a practical and scalable solution for texture-driven LULC classification in real-world scenarios involving limited spectral information or low-quantized data. Future work will focus on evaluating the performance of the proposed SVGM features on noisy datasets to better understand their behavior under challenging data conditions. We also plan to extend the comparison to more recent texture representations, including (a) modern handcrafted descriptors and (b) transfer-learning CNN-based texture features (e.g., VGG/ResNet-based descriptors and deep texture representations), which are known to be strong on texture datasets like DTD.

## Figures and Tables

**Table 1:** Classification results of the proposed SVGM compared with other methods for the AID Dataset.

Framework	Kernel	GLCM			LBP			CLBP			Proposed SVGM		
		OA (%)	AA (%)	KC	OA (%)	AA (%)	KC	OA (%)	AA (%)	KC	OA (%)	AA (%)	KC
Quantum SVM	QLinear	89.63	89.63	0.86	82.50	82.50	0.77	89.25	89.25	0.86	92.63	92.63	0.90
	QCircular	90.49	90.49	0.87	81.63	81.63	0.76	90.0	90.0	0.87	92.63	92.63	0.90
	QFull	92.5	92.5	0.91	83.38	83.38	0.78	90.63	90.63	0.88	92.63	92.63	0.90
Classical SVM	Poly	89.13	89.13	0.86	76.13	76.13	0.68	71.38	71.38	0.62	90.75	90.75	0.88
	RBF	89.88	89.88	0.87	76.63	76.63	0.69	76.25	76.25	0.68	91.63	91.63	0.89
DT	—	89.88	89.88	0.87	91.15	91.15	0.88	93.88	93.88	0.92	91.75	91.75	0.89
RF	—	92.75	92.75	0.90	92.38	92.38	0.90	94.13	94.13	0.92	93.75	93.75	0.92
1-D CNN	—	88.63	88.63	0.85	70.13	70.13	0.60	88.75	88.75	0.85	89.25	89.25	0.86
ANN	—	85.63	85.63	0.80	69.88	69.88	0.60	86.13	86.13	0.81	92.15	92.13	0.90

**Table 2:** Classification results of the proposed SVGM compared with other methods for the ALOT Dataset.

Framework	Kernel	GLCM			LBP			CLBP			Proposed SVGM		
		OA (%)	AA (%)	KC	OA (%)	AA (%)	KC	OA (%)	AA (%)	KC	OA (%)	AA (%)	KC
Quantum SVM	QLinear	92.0	92.0	0.89	67.63	67.63	0.57	66.5	66.5	0.56	94.38	94.38	0.93
	QCircular	92.0	92.0	0.89	72.0	72.0	0.63	67.0	67.0	0.56	94.0	94.0	0.92
	QFull	94.5	94.5	0.93	72.38	72.38	0.63	71.88	71.88	0.63	95.0	95.0	0.93
Classical SVM	Poly	88.13	88.13	0.84	60.13	60.13	0.47	52.88	52.88	0.37	90.75	90.75	0.88
	RBF	90.63	90.63	0.88	63.75	63.75	0.52	56.0	56.0	0.41	94.5	94.5	0.93
DT	—	95.88	95.88	0.95	82.88	82.88	0.77	88.5	88.5	0.85	97.13	97.13	0.96
RF	—	99.13	99.13	0.99	94.13	94.13	0.92	95.13	95.13	0.94	98.63	98.63	0.98
1-D CNN	—	88.25	88.25	0.84	64.0	64.0	0.52	68.38	68.38	0.58	89.87	89.87	0.87
ANN	—	87.75	87.75	0.84	50.25	50.25	0.34	64.5	64.5	0.53	89.13	89.13	0.86

**Table 3:** Classification result of the proposed SVGM compared with other methods for the Brodatz Dataset.

Framework	Kernel	GLCM			LBP			CLBP			Proposed SVGM		
		OA (%)	AA (%)	KC	OA (%)	AA (%)	KC	OA (%)	AA (%)	KC	OA (%)	AA (%)	KC
Quantum SVM	QLinear	81.5	81.5	0.75	74.13	74.13	0.66	74.0	74.0	0.65	81.38	81.38	0.81
	QCircular	82.38	82.38	0.77	75.5	75.5	0.67	75.13	75.13	0.67	81.63	81.63	0.76
	QFull	82.65	82.65	0.77	75.25	75.25	0.67	77.75	77.75	0.70	84.13	84.13	0.79
Classical SVM	Poly	77.87	77.87	0.71	64.75	64.75	0.53	65.25	65.25	0.54	79.88	79.88	0.73
	RBF	78.25	78.25	0.71	69.75	69.75	0.60	69.5	69.5	0.59	81.38	81.38	0.75
DT	—	83.25	83.25	0.78	84.5	84.5	0.79	83.25	83.25	0.78	85.5	85.5	0.81
RF	—	87.25	87.25	0.83	89.0	89.0	0.85	88.63	88.63	0.85	91.5	91.5	0.89
1-D CNN	—	79.38	79.38	0.73	70.5	70.5	0.61	67.63	67.63	0.57	82.63	82.63	0.77
ANN	—	75.63	75.63	0.68	68.88	68.88	0.59	67.25	67.25	0.56	75.75	75.75	0.68

**Table 4:** Classification result of the proposed SVGM compared with other methods for the DTD Dataset.

Framework	Kernel	GLCM			LBP			CLBP			Proposed SVGM		
		OA (%)	AA (%)	KC	OA (%)	AA (%)	KC	OA (%)	AA (%)	KC	OA (%)	AA (%)	KC
Quantum SVM	QLinear	98.5	98.5	0.98	86.25	86.25	0.82	94.38	94.38	0.93	99.0	99.0	0.99
	QCircular	98.5	98.5	0.98	86.5	86.5	0.82	93.0	93.0	0.91	99.0	99.0	0.99
	QFull	99.5	99.5	0.99	87.63	87.63	0.84	92.63	92.63	0.90	99.63	99.63	0.99
Classical SVM	Poly	98.5	98.5	0.98	76.75	76.75	0.69	79.25	79.25	0.72	98.0	98.0	0.97
	RBF	99.25	99.25	0.99	79.13	79.13	0.72	80.63	80.63	0.74	98.5	98.5	0.98
DT	—	99.37	99.37	0.99	96.5	96.49	0.95	96.0	96.0	0.95	98.63	98.63	0.98
RF	—	98.5	98.5	0.98	95.38	95.38	0.94	94.75	94.75	0.93	98.0	98.0	0.97
1-D CNN	—	97.38	97.38	0.97	80.88	80.88	0.75	77.13	77.13	0.70	92.0	91.99	0.89
ANN	—	94.5	94.5	0.93	76.65	76.65	0.69	81.13	81.13	0.75	96.5	96.5	0.95

**Table 5:** Classification result of the proposed SVGM compared with other methods for various texture patterns of the Desert Class of the AID Dataset.

Kernel	GLCM			LBP			CLBP			Proposed SVGM		
	OA (%)	AA (%)	KC	OA (%)	AA (%)	KC	OA (%)	AA (%)	KC	OA (%)	AA (%)	KC
QLinear	91.25	91.25	0.88	85.25	85.25	0.80	77.5	77.5	0.7	96.63	96.63	0.96
QCircular	91.5	91.5	0.89	84.75	84.75	0.80	77.5	77.5	0.7	96.75	96.75	0.96
QFull	92.25	92.25	0.90	84.75	84.75	0.80	79.5	79.5	0.73	96.38	96.38	0.95
Poly	90.01	90.01	0.87	79.25	79.25	0.72	75.0	75.0	0.68	93.13	93.13	0.91
RBF	90.5	90.5	0.87	79.13	79.13	0.72	75.5	75.5	0.67	94.5	94.5	0.93

**Table 6:** Effect of number of image patches used per class.

Kernel	Number of Image Patches															
	100				150				200				250			
	GLCM	LBP	CLBP	SVGM	GLCM	LBP	CLBP	SVGM	GLCM	LBP	CLBP	SVGM	GLCM	LBP	CLBP	SVGM
Poly	76.5	68.6	66.25	78.8	86.5	73.5	68.17	87.83	89.13	76.13	71.38	90.75	86.1	72.3	66.1	86.2
RBF	76.75	68.75	69.15	79.8	88.3	73.83	69.83	90.5	89.88	76.63	76.25	91.63	87.8	72.1	76.0	87.5
QLinear	76.5	66.75	71.13	80.13	89.17	80.17	86.67	91.5	89.63	82.5	89.25	92.63	90.2	81.7	85.8	87.9
QCircular	76.5	67.13	71.25	81.3	90.17	77.5	86.17	91.5	90.49	81.63	90.0	92.63	90.5	80.0	86.1	87.5
QFull	76.8	67.0	71.25	81.30	92.5	79.33	87.33	91.0	92.5	83.38	90.63	92.63	94.3	79.8	87.3	87.8

**Table 7:** Effect of the window size.

Kernel	Feature Used	Window Size			
		15	20	25	30
Poly	GLCM	84.5	87.5	89.13	85.2
	LBP	60.25	68.38	76.13	74.0
	CLBP	67.75	67.25	71.38	68.13
	SVGM	85.13	89.4	90.75	88.1
RBF	GLCM	85.75	88.5	89.87	86.7
	LBP	60.63	68.25	76.63	68.25
	CLBP	72.0	76.25	76.25	74.38
	SVGM	86.13	89.25	91.63	89.3
QLinear	GLCM	85.13	88.5	89.63	85.9
	LBP	74.5	82.38	82.5	80.4
	CLBP	84.25	88.5	89.25	81.25
	SVGM	86.38	91.88	92.63	89.6
QCircular	GLCM	85.75	89.38	90.49	87.4
	LBP	72.88	78.13	81.63	78.9
	CLBP	83.0	88.625	90.0	79.13
	SVGM	87.0	91.75	92.63	89.3
QFull	GLCM	88.13	91.25	92.5	88.1
	LBP	73.88	80.25	83.38	78.9
	CLBP	83.37	89.25	90.63	80.38
	SVGM	86.88	91.13	92.63	89.3

**Table 8:** Comparison of computational time.

Gray Level	Features Used			
	GLCM (ms)	LBP (ms)	CLBP (ms)	SVGM (ms)
8	588	1435	1543	87
16	797	1542	1582	87
32	990	1539	1603	87
64	1772	1568	1622	87

**Figure A1:**
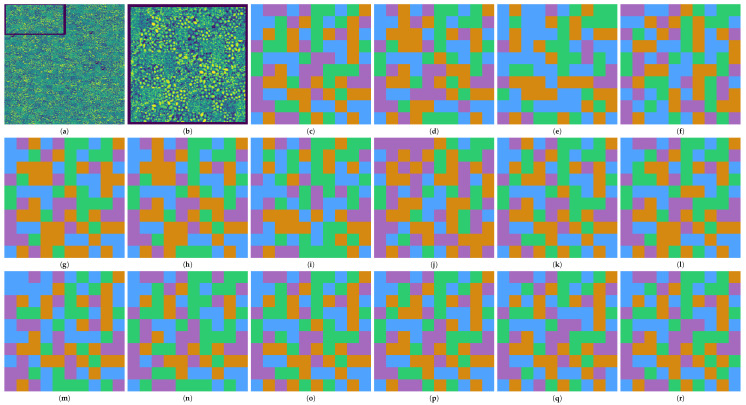

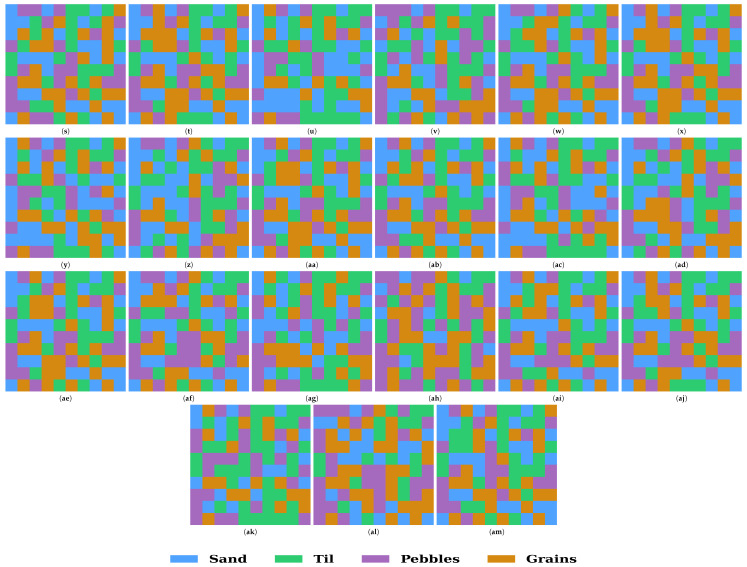
Classification maps produced by different classifiers trained using different feature sets like SVGM, GLCM, LBP, and CLBP on the ALOT dataset. (**a**) Collage Image, (**b**) Zoomed sub-image (**c**) Ground Truth (**d**) GLCM Poly (**e**) LBP Poly (**f**) CLBP Poly (**g**) SVGM Poly (**h**) GLCM RBF (**i**) LBP RBF (**j**) CLBP RBF (**k**) SVGM RBF (**l**) GLCM DT (**m**) LBP DT (**n**) CLBP DT (**o**) SVGM DT (**p**) GLCM RF (**q**) LBP RF (**r**) CLBP RF (**s**) SVGM RF (**t**) GLCM QLinear (**u**) LBP QLinear (**v**) CLBP QLinear (**w**) SVGM QLinear (**x**) GLCM QCircular (**y**) LBP QCircular (**z**) CLBP QCircular (**aa**) SVGM QCircular (**ab**) GLCM QFull (**ac**) LBP QFull (**ad**) CLBP QFull (**ae**) SVGM QFull (**af**) GLCM 1D-ANN (**ag**) LBP 1D-ANN (**ah**) CLBP 1D-ANN (**ai**) SVGM 1D-ANN (**aj**) GLCM CNN (**ak**) LBP CNN (**al**) CLBP CNN (**am**) SVGM CNN.

**Figure A2:**
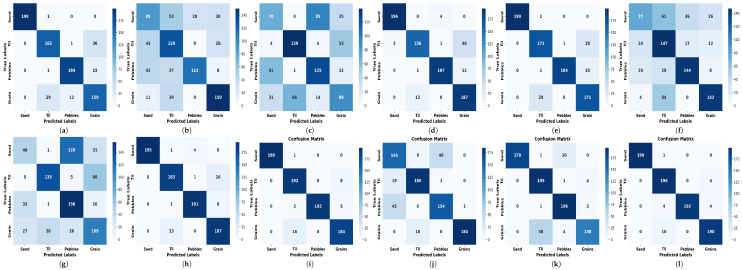

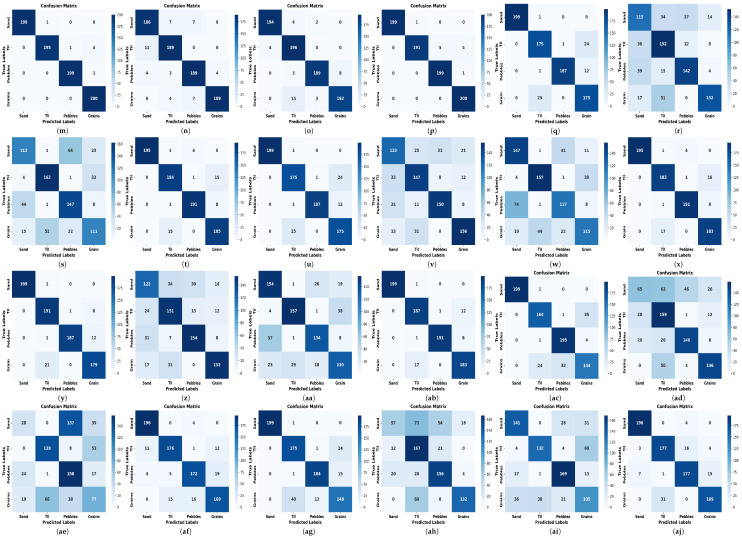
Confusion matrices depicting the performance of various classifiers on the ALOT dataset, illustrating the class-wise prediction performance of each model using different feature sets like SVGM, GLCM, LBP, and CLBP. (**a**) GLCM Poly (**b**) LBP Poly (**c**) CLBP Poly (**d**) SVGM Poly (**e**) GLCM RBF (**f**) LBP RBF (**g**) CLBP RBF (**h**) SVGM RBF (**i**) GLCM DT (**j**) LBP DT (**k**) CLBP DT (**l**) SVGM DT (**m**) GLCM RF (**n**) LBP RF (**o**) CLBP RF (**p**) SVGM RF (**q**) GLCM QLinear (**r**) LBP QLinear (**s**) CLBP QLinear (**t**) SVGM QLinear (**u**) GLCM QCircular (**v**) LBP QCircular (**w**) CLBP QCircular (**x**) SVGM QCircular (**y**) GLCM QFull (**z**) LBP QFull (**aa**) CLBP QFull (**ab**) SVGM QFull (**ac**) GLCM 1D-ANN (**ad**) LBP 1D-ANN (**ae**) CLBP 1D-ANN (**af**) SVGM 1D-ANN (**ag**) GLCM CNN (**ah**) LBP CNN (**ai**) CLBP CNN (**aj**) SVGM CNN.

**Figure A3:**
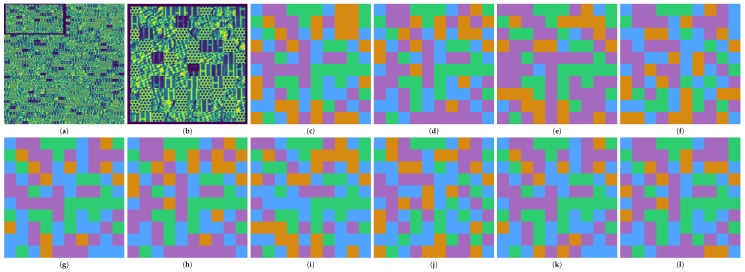

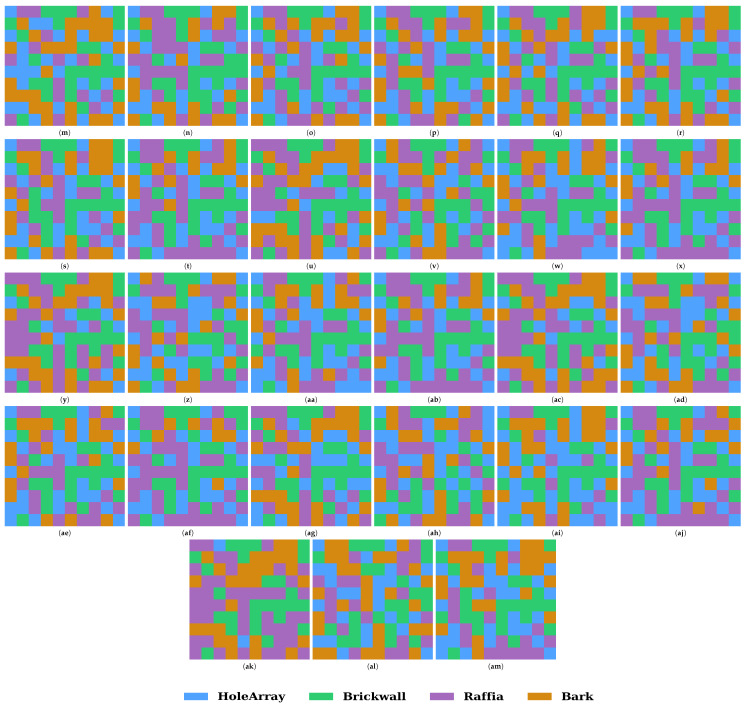
Classification maps produced by different classifiers trained using different feature sets like SVGM, GLCM, LBP, and CLBP on the Brodatz dataset. (**a**) Collage Image, (**b**) Zoomed sub-image (**c**) Ground Truth (**d**) GLCM Poly (**e**) LBP Poly (**f**) CLBP Poly (**g**) SVGM Poly (**h**) GLCM RBF (**i**) LBP RBF (**j**) CLBP RBF (**k**) SVGM RBF (**l**) GLCM DT (**m**) LBP DT (**n**) CLBP DT (**o**) SVGM DT (**p**) GLCM RF (**q**) LBP RF (**r**) CLBP RF (**s**) SVGM RF (**t**) GLCM QLinear (**u**) LBP QLinear (**v**) CLBP QLinear (**w**) SVGM QLinear (**x**) GLCM QCircular (**y**) LBP QCircular (**z**) CLBP QCircular (**aa**) SVGM QCircular (**ab**) GLCM QFull (**ac**) LBP QFull (**ad**) CLBP QFull (**ae**) SVGM QFull (**af**) GLCM 1D-ANN (**ag**) LBP 1D-ANN (**ah**) CLBP 1D-ANN (**ai**) SVGM 1D-ANN (**aj**) GLCM CNN (**ak**) LBP CNN (**al**) CLBP CNN (**am**) SVGM CNN.

**Figure A4:**
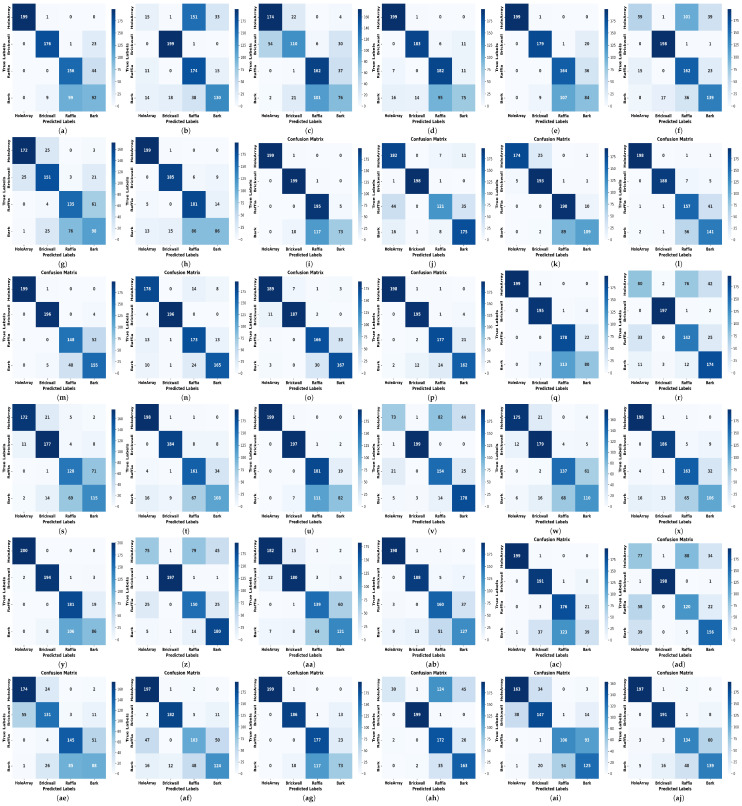
Confusion matrices depicting the performance of various classifiers on the Brodatz dataset, illustrating the class-wise prediction performance of each model using different feature sets—SVGM, GLCM, LBP, and CLBP. (**a**) GLCM Poly (**b**) LBP Poly (**c**) CLBP Poly (**d**) SVGM Poly (**e**) GLCM RBF (**f**) LBP RBF (**g**) CLBP RBF (**h**) SVGM RBF (**i**) GLCM DT (**j**) LBP DT (**k**) CLBP DT (**l**) SVGM DT (**m**) GLCM RF (**n**) LBP RF (**o**) CLBP RF (**p**) SVGM RF (**q**) GLCM QLinear (**r**) LBP QLinear (**s**) CLBP QLinear (**t**) SVGM QLinear (**u**) GLCM QCircular (**v**) LBP QCircular (**w**) CLBP QCircular (**x**) SVGM QCircular (**y**) GLCM QFull (**z**) LBP QFull (**aa**) CLBP QFull (**ab**) SVGM QFull (**ac**) GLCM 1D-ANN (**ad**) LBP 1D-ANN (**ae**) CLBP 1D-ANN (**af**) SVGM 1D-ANN (**ag**) GLCM CNN (**ah**) LBP CNN (**ai**) CLBP CNN (**aj**) SVGM CNN.

**Figure A5:**
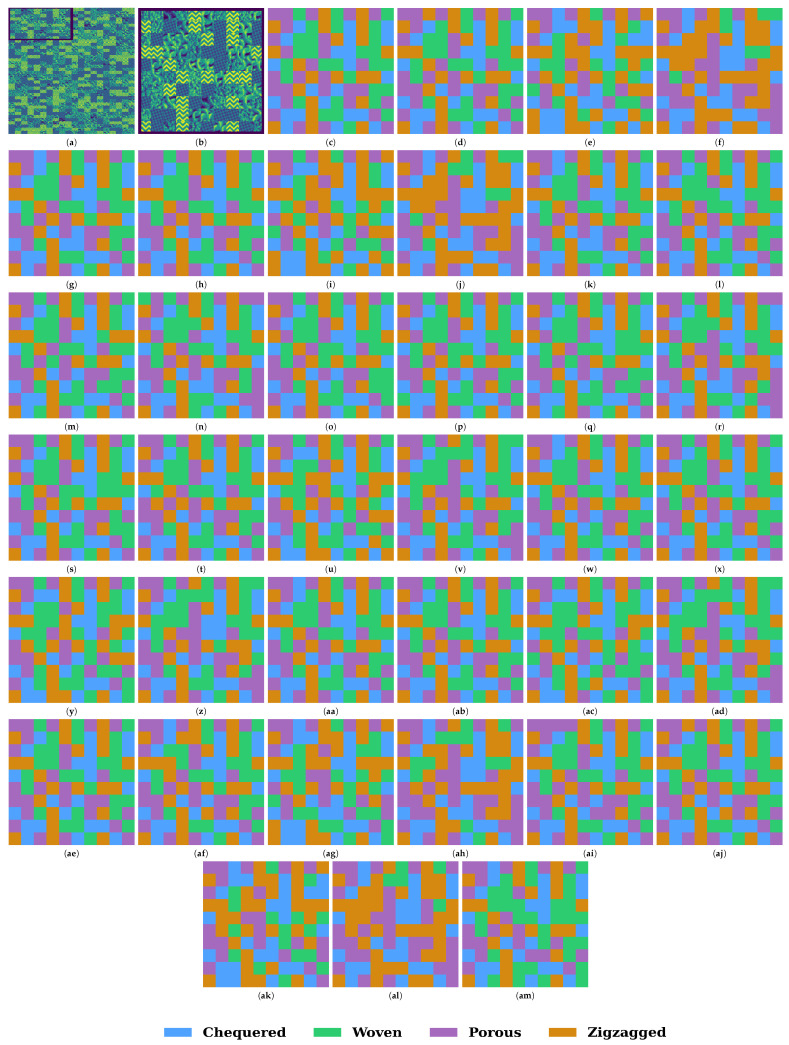
Classification maps produced by different classifiers trained using different feature sets like SVGM, GLCM, LBP, and CLBP on the DTD dataset. (**a**) Collage Image, (**b**) Zoomed sub-image (**c**) Ground Truth (**d**) GLCM Poly (**e**) LBP Poly (**f**) CLBP Poly (**g**) SVGM Poly (**h**) GLCM RBF (**i**) LBP RBF (**j**) CLBP RBF (**k**) SVGM RBF (**l**) GLCM DT (**m**) LBP DT (**n**) CLBP DT (**o**) SVGM DT (**p**) GLCM RF (**q**) LBP RF (**r**) CLBP RF (**s**) SVGM RF (**t**) GLCM QLinear (**u**) LBP QLinear (**v**) CLBP QLinear (**w**) SVGM QLinear (**x**) GLCM QCircular (**y**) LBP QCircular (**z**) CLBP QCircular (**aa**) SVGM QCircular (**ab**) GLCM QFull (**ac**) LBP QFull (**ad**) CLBP QFull (**ae**) SVGM QFull (**af**) GLCM 1D-ANN (**ag**) LBP 1D-ANN (**ah**) CLBP 1D-ANN (**ai**) SVGM 1D-ANN (**aj**) GLCM CNN (**ak**) LBP CNN (**al**) CLBP CNN (**am**) SVGM CNN.

**Figure A6:**
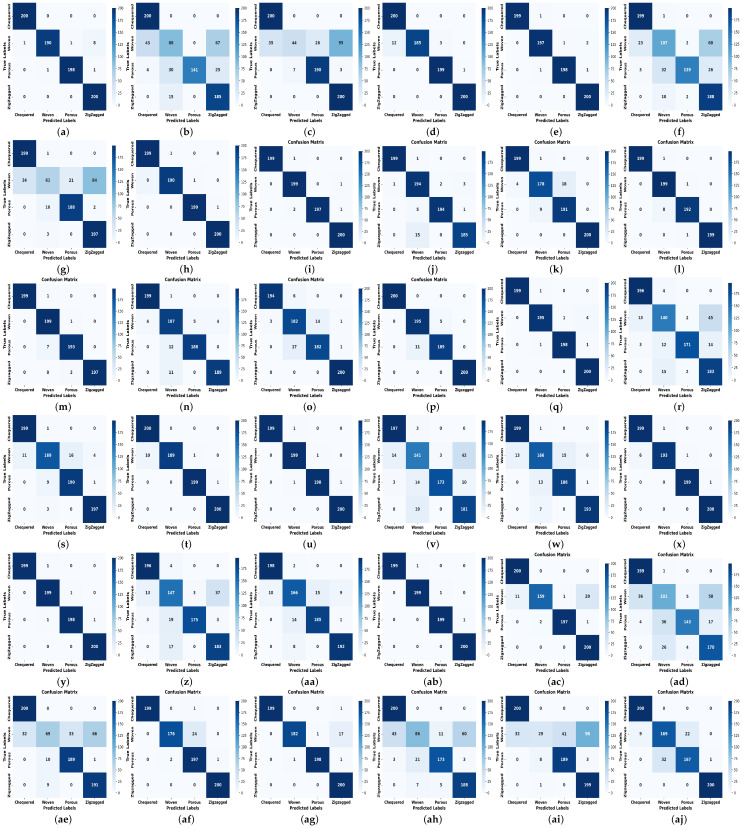
Confusion matrices depicting the performance of various classifiers on the DTD dataset, illustrating the class-wise prediction performance of each model using different feature sets SVGM, GLCM, LBP, and CLBP. (**a**) GLCM Poly (**b**) LBP Poly (**c**) CLBP Poly (**d**) SVGM Poly (**e**) GLCM RBF (**f**) LBP RBF (**g**) CLBP RBF (**h**) SVGM RBF (**i**) GLCM DT (**j**) LBP DT (**k**) CLBP DT (**l**) SVGM DT (**m**) GLCM RF (**n**) LBP RF (**o**) CLBP RF (**p**) SVGM RF (**q**) GLCM QLinear (**r**) LBP QLinear (**s**) CLBP QLinear (**t**) SVGM QLinear (**u**) GLCM QCircular (**v**) LBP QCircular (**w**) CLBP QCircular (**x**) SVGM QCircular (**y**) GLCM QFull (**z**) LBP QFull (**aa**) CLBP QFull (**ab**) SVGM QFull (**ac**) GLCM 1D-ANN (**ad**) LBP 1D-ANN (**ae**) CLBP 1D-ANN (**af**) SVGM 1D-ANN (**ag**) GLCM CNN (**ah**) LBP CNN (**ai**) CLBP CNN (**aj**) SVGM CNN.

## Data Availability

No new data were created or analyzed in this study. Data sharing is not applicable to this article.

## Abbreviations

The following abbreviations are used in this manuscript:

GLCM	Gray Level Co-Occurrence Matrix
LBP	Local Binary Pattern
CLBP	Complete LBP
SVM	Support Vector Machine
SVGM	Singular Values of GLCM
ANN	Artificial Neural Network
CNN	Convolutional Neural Network
QSVM	Quantum SVM
QC	Quantum Computer
SVD	Singular Value Decomposition
QML	Quantum Machine Learning
ALOT	Amsterdam Library of Textures Dataset
AID	Aerial Image Dataset
DTD	Describable Textures Dataset

## Appendix A. Quantum Computing Basics

Qubit is the smallest unit of information in quantum computing. A bit in classical computing can be in one of the two states, viz., 0 or 1. A qubit, when in 0 or 1 state, is called the basis state for a two-level system and is represented as

(A1) $$|0\rangle =\left[\begin{array}{c} 1\\ 0 \end{array}\right]\;\mathrm{and}\;|1\rangle =\left[\begin{array}{c} 0\\ 1 \end{array}\right]$$

If the qubit is in a linear combination of 0 and 1 then it is said to be in superposition and is represented as

(A2) |ϕ〉=α|0〉+β|1〉

where

(A3) $$|\phi \rangle =\left[\begin{array}{c} \alpha \\ \beta \end{array}\right]{,\;\mathrm{and}\;|\alpha |}^{2}+{\left|\beta \right|}^{2}=1$$

$\alpha^{2}\;\mathrm{and}\;\beta^{2}$ represent the probabilities of being in 0 and 1 state respectively, together preserving the law of probability. In general, a quantum system when in superposition is represented as, $|\phi \rangle =\sum_{i}^{N}C_{i}|i\rangle$ where $C_{i}$ is a complex number representing the amplitude of |i〉 a basis state in $N=2^{n}$ level system |ϕ〉 and $\sum_{i}C_{i}=1$. A classical register is capable of storing one of the $2^{n}$ possible combinations, whereas a quantum register can store all $2^{n}$ combinations spanned by ‘*n*’ qubits. An ‘*n*’ size quantum system is a collection of n qubits combined using a tensor product.

Each quantum state, when represented as a column vector, is called ‘ket’, and is symbolized as

$$|\phi \rangle =\left(\begin{array}{c} c_{1}\\ c_{2}\\ \ldots \\ c_{n} \end{array}\right)$$

and its complex conjugate, a row vector known as ‘bra’, is written as

$$\langle \phi |=\left(\begin{array}{c} c_{1}^{*},c_{2}^{*},\ldots ,c_{n}^{*} \end{array}\right).$$

They together form the mathematical ‘bracket’ named after the founder Paul Dirac.

In quantum computing, most operations are performed using gates. All quantum logic gates are reversible, unlike classical ones; hence, they have the same number of inputs and outputs. A gate that acts on ‘*n*’ qubits is represented by a $2^{n}\times 2^{n}$ matrix. The action of the gate on a specific quantum state is obtained by multiplying the corresponding vector and matrix, i.e., |ϕ〉, a vector which represents the quantum state, by the matrix ‘U’ representing the gate operation. Entanglement is a fundamental concept in quantum computing used to represent an inseparable product state. When qubits are entangled, measuring the state of one qubit swiftly determines the state of the other(s) when parted by large distances. Various types of entanglement can be created by first applying the Hadamard gate (H) and then the Controlled-X gate (CNOT) between the two qubits within a quantum circuit. This entanglement property is one of the key aspects that give quantum computing its power. Further information on quantum computing and its properties can be found in [70].

**Table A1:** Quantum gates [70,71].

Names	#Qubit	Symbol	Matrix	Gate Representation	Operation
Pauli X gate	1	X, $\sigma_{x}$	$\left(\begin{array}{cc} 0 & 1\\ 1 & 0 \end{array}\right)$		Inverts the amplitude of a quantum state
Pauli Y gate	1	Y, $\sigma_{y}$	$\left(\begin{array}{cc} 0 & -i\\ i & 0 \end{array}\right)$		Invert amplitude of Qubit and rotate phase vector of Qubit around y-axis
Pauli Z gate	1	Z, $\sigma_{z}$	$\left(\begin{array}{cc} 1 & 0\\ 0 & -1 \end{array}\right)$		Rotate phase of Qubit around z-axis by π
Hadamard gate	1	H	$\frac{1}{\sqrt{2}}\left(\begin{array}{cc} 1 & 1\\ 1 & -1 \end{array}\right)$		Used to put a quantum state into superposition
S gate	1	S,P	$\left(\begin{array}{cc} 1 & 0\\ 0 & i \end{array}\right)$		Rotate phase of Qubit when in state |1〉 by $\frac{\pi }{2}$ around z-axis
T gate	1	T	$\left(\begin{array}{cc} 1 & 0\\ 0 & e^{i\frac{\pi }{4}} \end{array}\right)$		Rotate phase of Qubit when in state |1〉 by $\frac{\pi }{4}$ around z-axis
CNOT gate	2	CNOT,CX	$\left(\begin{array}{cccc} 1 & 0 & 0 & 0\\ 0 & 1 & 0 & 0\\ 0 & 0 & 0 & 1\\ 0 & 0 & 1 & 0 \end{array}\right)$		Controlled NOT, i.e., flips the second qubit if the first qubit is 1
			$\left(\begin{array}{cccc} 1 & 0 & 0 & 0\\ 0 & 0 & 0 & 1\\ 0 & 0 & 1 & 0\\ 0 & 1 & 0 & 0 \end{array}\right)$		
CZ gate	2	CZ	$\left(\begin{array}{cccc} 1 & 0 & 0 & 0\\ 0 & 1 & 0 & 0\\ 0 & 0 & 1 & 0\\ 0 & 0 & 0 & -1 \end{array}\right)$		Controlled Z, i.e., applies **Z** gate on the second qubit if the first qubit is 1
Swap gate	2	SWAP	$\left(\begin{array}{cccc} 1 & 0 & 0 & 0\\ 0 & 0 & 1 & 0\\ 0 & 1 & 0 & 0\\ 0 & 0 & 0 & 1 \end{array}\right)$		Swaps the two states

## References

[1] Bu H.H., Kim N.C., Kim S.H. (2023). Content-based image retrieval using a fusion of global and local features. ETRI J..

[2] Ghalati M.K., Nunes A., Ferreira H., Serranho P., Bernardes R. (2021). Texture analysis and its applications in biomedical imaging: A survey. IEEE Rev. Biomed. Eng..

[3] Nisbett W.H., Kavuri A., Das M. (2020). On the correlation between second order texture features and human observer detection performance in digital images. Sci. Rep..

[4] Zhu D., Pan R., Gao W., Zhang J. (2015). Yarn-dyed fabric defect detection based on autocorrelation function and GLCM. Autex Res. J..

[5] Islam R., Uddin J., Kim J.M. (2018). Texture analysis based feature extraction using Gabor filter and SVD for reliable fault diagnosis of an induction motor. Int. J. Inf. Technol. Manag..

[6] Quintero-Rincón A., Di-Pasquale R., Quintero-Rodríguez K., Batatia H. (2025). Computer-based quantitative image texture analysis using multi-collinearity diagnosis in chest X-ray images. PLoS ONE.

[7] Hossain M.S., Basak N., Mollah M.A., Nahiduzzaman M., Ahsan M., Haider J. (2025). Ensemble-based multiclass lung cancer classification using hybrid CNN-SVD feature extraction and selection method. PLoS ONE.

[8] Cáceres-Tello J., Galán-Hernández J.J. (2025). Mathematical Evaluation of Classical and Quantum Predictive Models Applied to PM2.5 Forecasting in Urban Environments. Mathematics.

[9] Mohammadpour P., Viegas D.X., Viegas C. (2022). Vegetation mapping with random forest using sentinel 2 and GLCM texture feature—A case study for Lousã region, Portugal. Remote Sens..

[10] Andrearczyk V., Whelan P.F. (2016). Using filter banks in Convolutional Neural Networks for texture classification. Pattern Recognit. Lett..

[11] Puissant A., Hirsch J., C. W. (2005). The utility of texture analysis to improve per-pixel classification for high to very high spatial resolution imagery. Int. J. Remote Sens..

[12] Zhou J., Yan Guo R., Sun M., Di T.T., Wang S., Zhai J., Zhao Z. (2017). The Effects of GLCM parameters on LAI estimation using texture values from Quickbird Satellite Imagery. Sci. Rep..

[13] De Siqueira F.R., Schwartz W.R., Pedrini H. (2013). Multi-scale gray level co-occurrence matrices for texture description. Neurocomputing.

[14] Humeau-Heurtier A. (2019). Texture feature extraction methods: A survey. IEEE Access.

[15] Ramola A., Shakya A.K., Van Pham D. (2020). Study of statistical methods for texture analysis and their modern evolutions. Eng. Rep..

[16] Kabir S., He D.C., Sanusi M.A., Wan Hussina W.M.A. (2010). Texture analysis of IKONOS satellite imagery for urban land use and land cover classification. Imaging Sci. J..

[17] Sun C., Wen Z. (2021). Research Progress in Remote Sensing Image Texture Analysis. J. Phys. Conf. Ser..

[18] Bharati M.H., Liu J., MacGregor J. (2004). Image texture analysis: Methods and comparisons. Chemom. Intell. Lab. Syst..

[19] Chaurasia K., Garg P.K. (2013). A brief review on texture analysis methods. Stud. Surv. Mapp. Sci..

[20] Saboori M., Asghar Torahi A., Reza Riyahi Bakhtyari H. (2019). Combining multi-scale textural features from the panchromatic bands of high spatial resolution images with ANN and MLC classification algorithms to extract urban land uses. Int. J. Remote Sens..

[21] Haralick R.M., Shanmugam K., Dinstein I.H. (1973). Textural Features for Image Classification. IEEE Trans. Syst. Man Cybern..

[22] Mhangara P., Odindi J. (2013). Potential of texture-based classification in urban landscapes using multispectral aerial photos. S. Afr. J. Sci..

[23] Kupidura P. (2019). The Comparison of Different Methods of Texture Analysis for Their Efficacy for Land Use Classification in Satellite Imagery. Remote Sens..

[24] Jan Y.K., Hung I.Y.J., Cheung W.C. (2025). Texture Analysis in Musculoskeletal Ultrasonography: A Systematic Review. Diagnostics.

[25] Shaban M.A., Dikshit O. (2001). Improvement of classification in urban areas by the use of textural features: The case study of Lucknow city, Uttar Pradesh. Int. J. Remote Sens..

[26] Gong P., Marceau D.J., Howarth P.J. (1992). A comparison of spatial feature extraction algorithms for land-use classification with SPOT HRV data. Remote Sens. Environ..

[27] Suresh A., Shunmuganathan K.L. (2012). Image texture classification using gray level co-occurrence matrix based statistical features. Eur. J. Sci. Res..

[28] Marceau D., Howarth P.J., Dubois J., Gratton D. (1990). Evaluation Of The Grey-level Co-occurrence Matrix Method For Land-cover Classification Using Spot Imagery. IEEE Trans. Geosci. Remote Sens..

[29] Zhang X., Cui J., Wang W., Lin C. (2017). A Study for Texture Feature Extraction of High-Resolution Satellite Images Based on a Direction Measure and Gray Level Co-Occurrence Matrix Fusion Algorithm. Sensors.

[30] Chakraborty P., Yousuf M.A., Islam S., Khatun M., Sarker A., Rahman S. (2022). Analysis of Texture Feature Extraction Technique in Image Processing. Distributed Sensing and Intelligent Systems: Proceedings of ICDSIS 2020.

[31] Wu Y., Lou L., Wang J. (2022). Cotton fabric defect detection based on K-SVD dictionary learning. J. Nat. Fibers.

[32] Xu M., Yu Q., Chen S., Lin J. (2024). Rolling Bearing Fault Diagnosis Based on CNN-LSTM with FFT and SVD. Information.

[33] Patil S.H., Kulkarni R. (2022). Surface roughness measurement based on singular value decomposition of objective speckle pattern. Opt. Lasers Eng..

[34] Basha S.A., Kalyani V.K., Sai Hemanth D.T. (2024). SVD-Based Method for High-Fidelity Color to Grayscale Image Conversion. Emerging Technologies in Computing.

[35] Selvan S., Ramakrishnan S. (2007). SVD-Based Modeling for Image Texture Classification Using Wavelet Transformation. IEEE Trans. Image Process..

[36] Ramakrishnan S., Selvan S. (2007). Multiwavelets domain singular value features for image texture classification. J. Zhejiang Univ.-Sci. A.

[37] Qiao Y.L., Zhao Y., Song C.Y., Zhang K.G., Xiang X.Z. (2021). Graph wavelet transform for image texture classification. IET Image Process..

[38] Nagarajan B., Devendran V. (2012). Vehicle Classification under Cluttered Background and Mild Occlusion Using Zernike Features. Procedia Eng..

[39] Arora P., Mehta R., Soni P. (2026). Multimodal medical image analysis using deep learning registration and LWT-SVD fusion. Discov Comput..

[40] Havlíček V., Córcoles A.D., Temme K., Harrow A.W., Kandala A., Chow J.M., Gambetta J.M. (2019). Supervised learning with quantum-enhanced feature spaces. Nature.

[41] Shaik R.U., Periasamy S. (2022). Accuracy and processing speed trade-offs in classical and quantum SVM classifier exploiting PRISMA hyperspectral imagery. Int. J. Remote Sens..

[42] Saini S., Khosla P.K., Kaur M., Singh G. (2020). Quantum Driven Machine Learning. Int. J. Theor. Phys..

[43] Delilbasic A., Cavallaro G., Willsch M., Melgani F., Riedel M., Michielsen K. Quantum Support Vector Machine Algorithms for Remote Sensing Data Classification. Proceedings of the 2021 IEEE International Geoscience and Remote Sensing Symposium IGARSS.

[44] Rebentrost P., Mohseni M., Lloyd S. (2014). Quantum Support Vector Machine for Big Data Classification. Phys. Rev. Lett..

[45] Schuld M., Killoran N. (2019). Quantum Machine Learning in Feature Hilbert Spaces. Phys. Rev. Lett..

[46] Hidalgo M.C. (2025). Comparative Analysis of a Quantum SVM with an Optimized Kernel Versus Classical SVMs. IEEE Access.

[47] Slabbert D., Petruccione F. (2025). Classical-quantum approach to image classification: Autoencoders and quantum SVMs. AVS Quantum Sci..

[48] Radhi E.A., Kamil M.Y., Mohammed M.A. (2025). Quantum Machine and Deep Learning for Medical Image Classification: A Systematic Review of Trends, Methodologies, and Future Directions. Iraqi J. Comput. Sci. Math..

[49] Miroszewski A., Mielczarek J., Czelusta G., Szczepanek F., Grabowski B., Le Saux B., Nalepa J. (2023). Detecting clouds in multispectral satellite images using quantum-kernel support vector machines. IEEE J. Sel. Top. Appl. Earth Obs. Remote Sens..

[50] IBM Corporation (2018). Real Quantum Computers. Right at Your Fingertips. IBM. https://quantum.cloud.ibm.com/signin.

[51] Pai A.G., Buddhiraju K.M., Durbha S.S. (2022). Multiclass classification of hyperspectral remote sensed data using QSVC. Remote Sensing for Agriculture, Ecosystems, and Hydrology XXIV.

[52] Pai A.G., Buddhiraju K.M., Durbha S.S. Texture Based LULC Classification of Images Using QSVM. Proceedings of the IGARSS 2023—2023 IEEE International Geoscience and Remote Sensing Symposium.

[53] Pai A.G., Buddhiraju K.M., Durbha S.S. Binary Classification of Remotely Sensed Images Using SVD Based GLCM Features in Quantum Framework. Proceedings of the IGARSS 2024—2024 IEEE International Geoscience and Remote Sensing Symposium.

[54] D-Wave Systems Inc (2022). Begin Your Quantum Journey. D-Wave. https://www.dwavesys.com/learn/quantum-computing.

[55] Delilbasic A., Le Saux B., Riedel M., Michielsen K., Cavallaro G. (2023). A single-step multiclass SVM based on quantum annealing for remote sensing data classification. IEEE J. Sel. Top. Appl. Earth Obs. Remote Sens..

[56] Otgonbaatar S., Kranzlmüller D. (2023). Exploiting the Quantum Advantage for Satellite Image Processing: Review and Assessment. IEEE Trans. Quantum Eng..

[57] Otgonbaatar S., Datcu M. (2021). Assembly of a Coreset of Earth Observation Images on a Small Quantum Computer. Electronics.

[58] Xia G.S., Hu J., Hu F., Shi B., Bai X., Zhong Y., Zhang L., Lu X. (2017). AID: A Benchmark Data Set for Performance Evaluation of Aerial Scene Classification. IEEE Trans. Geosci. Remote Sens..

[59] Cimpoi M., Maji S., Kokkinos I., Mohamed S., Vedaldi A. Describing Textures in the Wild. Proceedings of the IEEE Conference on Computer Vision and Pattern Recognition (CVPR).

[60] Weber A.G. (2018). The USC-SIPI Image Database: Version 6. http://sipi.usc.edu/database.

[61] Hersey I. (1968). Textures: A photographic album for artists and designers by phil brodatz. Leonardo.

[62] Burghouts G.J., Geusebroek J.M. (2009). Material-Specific Adaptation of Color Invariant Features. Pattern Recognit. Lett..

[63] Otsu N. (1979). A Threshold Selection Method from Gray-Level Histograms. IEEE Trans. Syst. Man, Cybern..

[64] IBM (2018). ZZ Feature Map. https://quantum.cloud.ibm.com/docs/en/api/qiskit/qiskit.circuit.library.ZZFeatureMap.

[65] Ojala T., Pietikäinen M., Harwood D. (1996). A comparative study of texture measures with classification based on featured distributions. Pattern Recognit..

[66] Ojala T., Pietikainen M., Maenpaa T. (2002). Multiresolution gray-scale and rotation invariant texture classification with local binary patterns. IEEE Trans. Pattern Anal. Mach. Intell..

[67] Guo Z., Zhang L., Zhang D. (2010). A Completed Modeling of Local Binary Pattern Operator for Texture Classification. IEEE Trans. Image Process..

[68] Zhao G., Wu G., Liu Y., Chen J. Texture Classification Based on Completed Modeling of Local Binary Pattern. Proceedings of the 2011 International Conference on Computational and Information Sciences.

[69] Cimpoi M., Maji S., Vedaldi A. Deep filter banks for texture recognition and segmentation. Proceedings of the IEEE Conference on Computer Vision and Pattern Recognition.

[70] Nielsen M., Chuang I. (2010). Quantum Computation and Quantum Information: 10th Anniversary Edition.

[71] Williams C.P., Clearwater S.H. (1997). Explorations in Quantum Computing.

